# Building the continuum of competency-based medical education

**DOI:** 10.1007/s40037-015-0191-y

**Published:** 2015-07-17

**Authors:** William F. Iobst, Eric S. Holmboe

**Affiliations:** 1 Commonwealth Medical College, Scranton, PA USA; 2 Accreditation Council for Graduate Medical Education, Chicago, IL USA

Paul Batalden is credited with the observation that, ‘every system is perfectly designed for the results it generates’. In this volume of Perspectives in Medical Education, Chen and associates highlight the importance of this observation by describing self-reported gaps in preparedness of learners transitioning from undergraduate to graduate medical training [1]. By identifying these gaps, these authors highlight one of the most serious challenges facing the medical education community as it operationalizes competency-based medical education. Using Batalden’s axiom, learners experience a medical education system that is perfectly designed to inadequately prepare them for the next stage of their professional development and ultimately to work competently in the health care delivery system of the future.

For over 100 years, medical education has been delivered along a continuum that can be characterized as a series of linked, but independent silos. While the design of these silos may vary worldwide, they typically include premedical education, undergraduate medical education, graduate medical education and continuing medical education. Historically, the alignment of learning outcomes across these silos has reflected a confederacy rather than a union of stakeholders. Recognizing this reality, in 2010, the Carnegie Foundation called for a unified roadmap for medical education across this continuum [2]. Key elements of that roadmap included standardized core learning outcomes, flexibility to allow the achievement of competency milestones at variable rates, the establishment of effective mechanisms to coordinate standards and the establishment of rigorous and progressively higher levels of competency.

In spite of this and other calls to action, the education community has just begun the work of describing meaningful developmental outcomes for each educational silo and has yet to effectively integrate this work across the professional career of a physician [3]. The gaps reported by Chen are not unique. Studies by Crosson and Mattar have identified similar deficiencies in resident and fellow competence at significant transitions including the transition to unsupervised practice [4, 5]. Crosson specifically commented, ‘educators, accrediting bodies and other stakeholders will need to work together to clarify gaps, prioritize them and determine which can best be addressed in medical school, residency, fellowship or clinical practice’. Given what we know about the limitations of self-assessment, and despite the low response rate observed in the Chen study, the fact that students report feeling ill-prepared in many of the core competencies needed for current and future practice is striking.

The development of milestones that define the ACGME and CanMeds competencies and entrustment-based assessment utilizing the concept of entrustable professional activities (EPAs) have begun to better define this continuum [6–8]. As granular descriptions of the knowledge, skills and attitudes/ behaviours that define the competencies, milestones provide a shared understanding of competence, enhance feedback and provide greater uniformity when making promotion decisions [9, 10]. Entrustment-based assessments focus on the actual work of patient care: those discrete activities that all physicians are trusted to do. While still nascent, core EPAs at the transition from undergraduate to graduate training and beyond have already been proposed [11, 12]. However, because future physicians will need to deliver safe and effective patient care that achieves the Institute of Health Care Improvement Triple Aim, a number of additional steps must occur in the evolution of the medical education continuum [13].

1. A common framework and language of competency that spans the continuum of the medical profession must be embraced by medical education stakeholders. The significant progress made advancing the framework of competency through the definition of general competencies, milestones and assessment strategies, including the use of entrustment, must be harmonized across the entire continuum of professional development. Competency frameworks such as the ACGME General Competencies and the CanMeds Competencies have been embraced by many, but not all key stakeholders. To truly standardize expectations across the continuum of medical education, all medical educators, accreditors and certifiers must agree to common frameworks and expected outcomes of training.
2. The role of the student in the development and progression of their competence must be clearly defined. As early as 2002, Carraccio and associates highlighted that learners must be active participants in the competency-based educational process [14]. To best define the proximate zone of learning, that ideal space where the learner is most able to engage learning for excellence, students must be actively engaged in self-directed assessment that advances their progress towards achieving ongoing competence [15]. However, as Davis and others have written, self-assessment without the guidance of an external reference and coach is suboptimal [16]. Appropriately activated learners facilitate learning and this removes ambiguity regarding expected outcomes.
3. Medical educators must also appreciate the complexity associated with the transition from the traditional time-based process model of medical education to one that is competency driven. This transition will need to be evolutionary and iterative. Developing a granular description of the knowledge, skills and attitudes (milestones) that define the general competencies highlights this evolution as does the use of EPAs as an assessment strategy to generate robust and rigorous holistic data that inform decisions about competence in the actual delivery of patient care.
4. Finally, the learning continuum must balance the need for robust experiential learning with the delivery of safe and effective patient care. This requires appropriate supervision of learners at every stage of their development. Kogan and associates have proposed that these two needs can be achieved by balancing the known competence of the learner through robust assessment with a level of supervision that always ensures safety in the clinical encounter [17]. Effective application of this principle at each stage of a learner’s development can provide a mechanism for stimulating the developmental progression of competency at all stages of the continuum. However, to determine the appropriate level of needed supervision, assessment must accurately determine what a learner can be entrusted to do. This decision will be dynamic and unique to the context of the clinical event and will require accurate work-based assessment. To complete this task, faculty will need a robust tool box of assessments and even more importantly will need to develop a shared understanding of the strengths and weaknesses of each tool in the box. This will not happen spontaneously and will require robust and effective faculty development.

Policymakers, educators and now learners have spoken: the current system is not operating at a high level of effectiveness. It is now time to apply the same creative energy that produced competency-based milestones and entrustments to the work of defining an integrated continuum of medical education.
